# Epidemiology of penile cancer in Poland compared to other European countries

**DOI:** 10.1002/cam4.70092

**Published:** 2024-08-20

**Authors:** Iwona Wnętrzak, Mateusz Czajkowski, Klaudia Barańska, Marta Miklewska, Urszula Wojciechowska, Roman Sosnowski, Joanna A. Didkowska

**Affiliations:** ^1^ Department of General and Oncological Urology Praski Hospital Warsaw Poland; ^2^ Department of Urology Medical University of Gdańsk Gdańsk Poland; ^3^ Polish National Cancer Registry Maria Sklodowska‐Curie National Research Institute of Oncology Warsaw Poland; ^4^ Faculty of Biomedical Engineering Silesian University of Technology Zabrze Poland; ^5^ Department of Dietetics, Institute of Human Nutrition Sciences Warsaw University of Life Sciences Warsaw Poland; ^6^ Department of Urology and Oncological Urology MSWiA Hospital, Warmian‐Masurian Cancer Center Olsztyn Poland; ^7^ Department of Epidemiology and Cancer Prevention Maria Sklodowska‐Curie National Research Institute of Oncology Warsaw Poland

**Keywords:** epidemiology, incidence, mortality, penile neoplasms

## Abstract

**Objectives:**

To examine the epidemiology of penile cancer in Poland compared to other European countries.

**Materials and Methods:**

Incidence and mortality data were acquired from the national cancer registries in Europe and WHO Mortality Database, respectively. The data are presented as age‐standardised morbidity and mortality rates, calculated according to the standard population of the world. We utilised Joinpoint analysis to assess the trends in morbidity and mortality and calculated the average rate of increase or decrease (Annual Percentage Change, Average Annual Percentage Change). Additionally, we estimate the proxy survival rates for each country.

**Results:**

Our study is the first to cover the incidence of penile cancer in many European countries and estimates an approximate survival rate for large populations, which is rarely cited in the literature. The 40+ age group presented graphically in the article covered more than 90% of penile cancer cases and deaths. In the countries examined, there was an excess of deaths over incidence in the oldest age groups (75 years or older). Poland had intermediate incidence and mortality rates.

**Conclusions:**

Unlike many European countries, Poland is witnessing an increasing trend of penile cancer mortality. The higher death toll among those aged 75 years or older may suggest a lack of recognition of cancer symptoms and inadequate attention to elderly patients by the healthcare system. There is also evidence of underreporting penile cancer cases. Establishing centralised healthcare systems for rare cancers is a commendable development that should be emulated by other European countries, including Poland.

## 1 INTRODUCTION

This study was inspired by the article Global Pattern and Trends in Penile Cancer Incidence: Population‐Based Study published in the JMIR Public Health and Surveillance in 2022. Referring to mentioned article the Poland's position based only on data obtained from the registration office in Kielce, which is part of the Polish National Cancer Registry (PNCR). These data, covering only 3.2% of the Polish population, cannot constitute a reliable source and representative sample of penile cancer epidemiology in Poland. Moreover, it also refers to the Cancer Incidence in Five Continents.^1^ They concerned only 26% of the Polish population in the years 2008–2012 (including data from selected regions of Poland: Świętokrzyskie, Wielkopolskie, Dolnośląskie, Lubelskie and Podkarpackie). In our study, we extended the analysis to the years 1999–2020.

Penile cancer is considered a rare disease in the European Union, as it affects fewer than one person per 2000 cases. The incidence of penile cancer in highly developed countries remains at a level of 0.1–1 per 100,000 men, which is equivalent to approximately 0.002–0.02 for 2000 men.^2^ In 2020, the age‐standardised incidence rate for penile cancer worldwide was estimated to be 0.8 per 100,000 men, and the age‐standardised mortality rate was 0.29 per 100,000 men, resulting in 36,068 incident cases and 13,211 deaths.^3^

This study aimed to examine the epidemiology of penile cancer in Poland compared to other European countries. The selection of Poland as the subject of this study is appropriate due to the increasing incidence and exceptionally high mortality rate of penile cancer in the country.

## 2 MATERIALS AND METHODS

Incidence data were obtained from national cancer registers in Europe (Poland—PNCR, https://onkologia.org.pl/. Czechia–Epidemiology of Malignant Tumours in the Czech Republic, https://www.svod.cz/. Belgium–Belgian Cancer Registry, https://kankerregister.org/. Germany–Krebsdaten, https://www.krebsdaten.de/. Estonia–Statistics Estonia, https://andmed.stat.ee/en/stat. Finland–Finnish Cancer Registry, https://cancerregistry.fi/information/. Ireland–National Cancer Registry Ireland, https://www.ncri.ie/. Netherlands–the Netherlands Comprehensive Cancer Organisation https://iknl.nl/en; Austria, Bulgaria, Latvia, Lithuania, Slovenia, Switzerland—on request). To analyse mortality in European countries (27 European countries), we used data from the WHO Mortality Database https://www.who.int/data/data‐collection‐tools/who‐mortality‐database. For Poland, we conducted the analysis based on data from the PNCR covering the years 1999–2020. Table 1 presents the range of available data on morbidity and mortality in each country. Data from Switzerland were aggregated into 4‐year groups (1995–1999, 2000–2004, 2005–2009, 2010–2014, 2015–2019). Data directly from the Nordcan database (Scandinavian countries) did not cover the single ICD‐10 code for penile cancer, C60. Nordcan provides data for combined C60 + C63 only, that is, penile cancer and malignant cancer of other unspecified male genital organs. The data for Finland were downloaded directly from the Finnish Register. In other countries, data were available only on request or grouped as C60 + C63.

**TABLE 1:** Years of observation of each country.

Country	Beginning of observation—morbidity	End of observation—morbidity	Beginning of observation—mortality	End of observation—mortality
Austria	1999	2020	2002	2020
Belgium	2004	2020	1999	2018
Bulgaria	1999	2020	2005	2019
Croatia	N/A	1999	2019	
Czechia	1999	2020	1999	2020
Denmark	N/A	1999	2018	
Estonia	1999	2020	1999	2015
Finland	1999	2020	1999	2019
France	N/A	2000	2017	
Germany	1999	2019	1999	2020
Greece	N/A	2014	2019	
Hungary	N/A	1999	2019	
Ireland	1999	2020	2007	2018
Italy	N/A	2003	2017	
Latvia	1999	2017	1999	2020
Lithuania	1999	2019	1999	2020
Netherlands	1999	2020	1999	2020
Norway	N/A	1999	2016	
Poland	1999	2020	1999	2020
Portugal	N/A	2002	2018	
Romania	N/A	1999	2019	
Slovakia	N/A	1999	2019	
Slovenia	1999	2019	1999	2020
Spain	N/A	1999	2020	
Sweden	N/A	1999	2018	
Switzerland	1995	2019	1999	2019
United Kingdom	N/A	2001	2020	

All data are presented as age‐standardised morbidity and mortality rates according to the standard population of the world (ASW).

**FIGURE 1:**
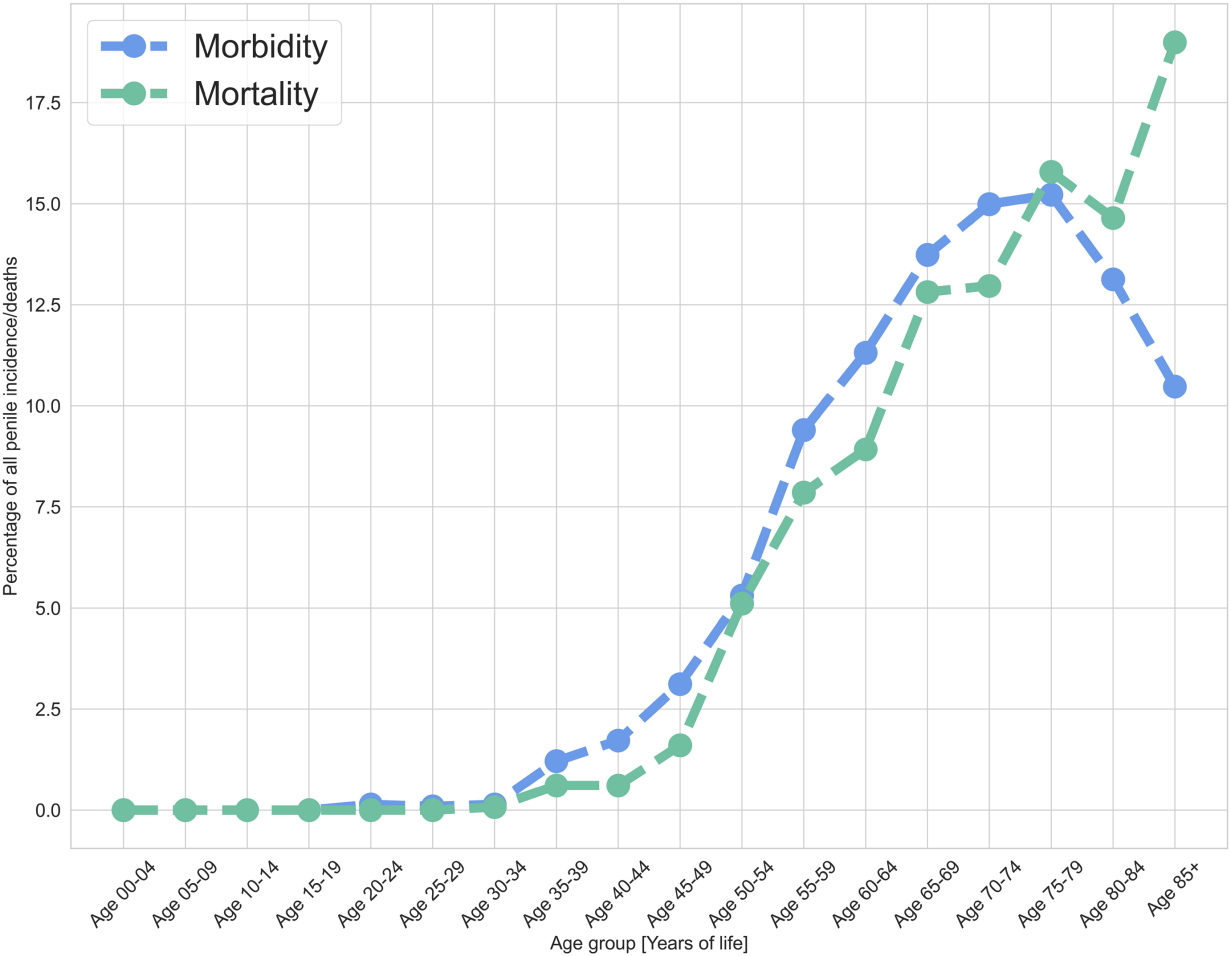
Percentage of deaths and incidence in each age group.

The 40+ age group presented graphically in the article was distinguished based on age‐specific morbidity and mortality curves and covered over 90% of penile cancer cases and deaths (Figure 1). Two countries were excluded from the incidence age group analysis: Finland and the Netherlands (data without the appropriate age groups).

**FIGURE 2:**
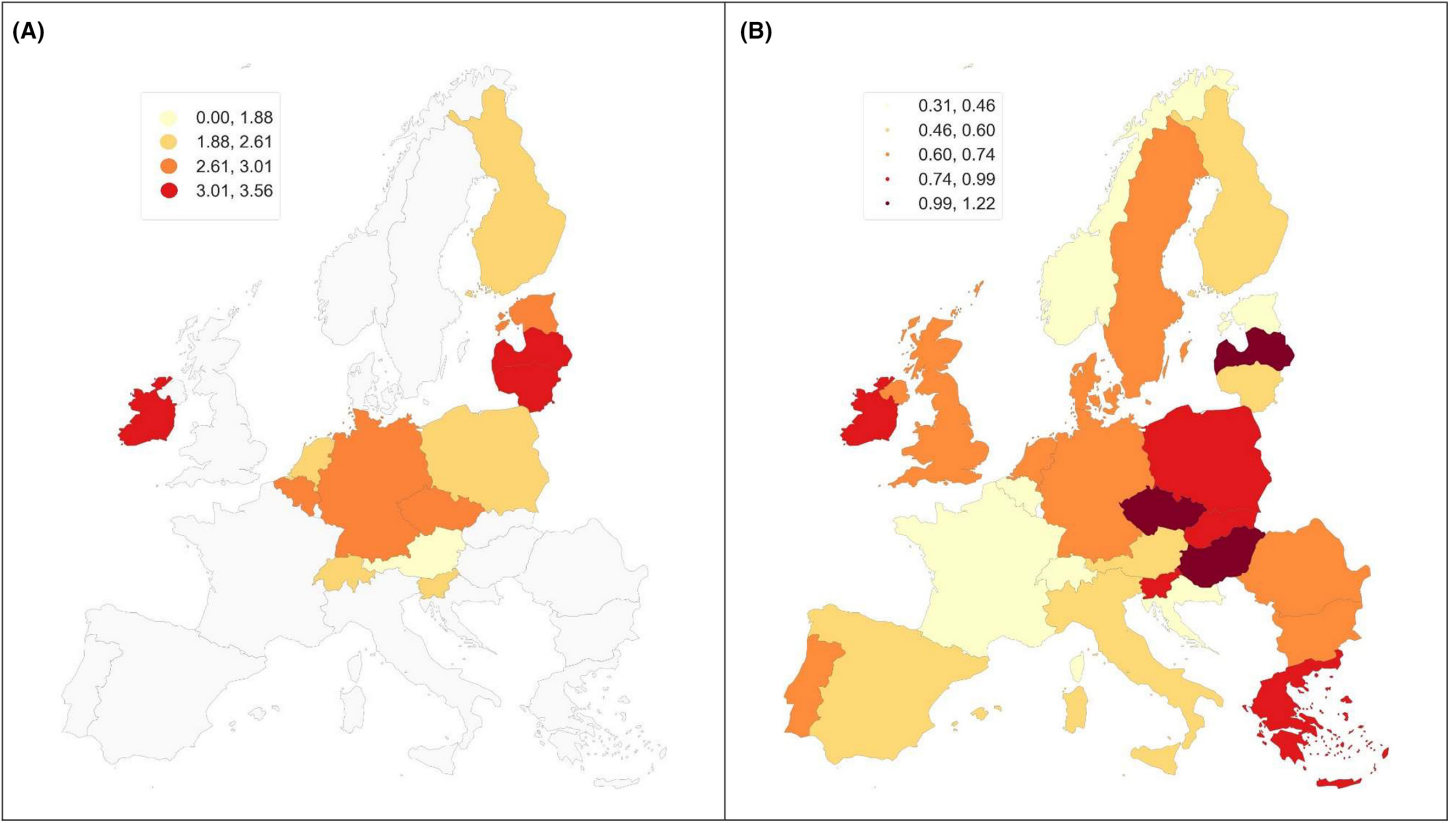
European map of penile cancer morbidity (A) and mortality (B) for age group 40+.

The study presents morbidity and mortality maps for the 40+ age group (Figure 2). The map of Poland divided into voivodeships comes from Geoportal, https://www.geoportal.gov.pl/. provided by GIS‐Support https://gis‐support.pl/. Analyses were performed in Python, with particular use of the Geopandas package https://geopandas.org/en/stable/index.html. The gradation used in the maps was based on the Fisher‐Jenks method of natural divisions, maximising the variance between classes and minimising the variance within classes. The maps present data for the most recent observed year for a particular country (Table 1).

**FIGURE 3:**
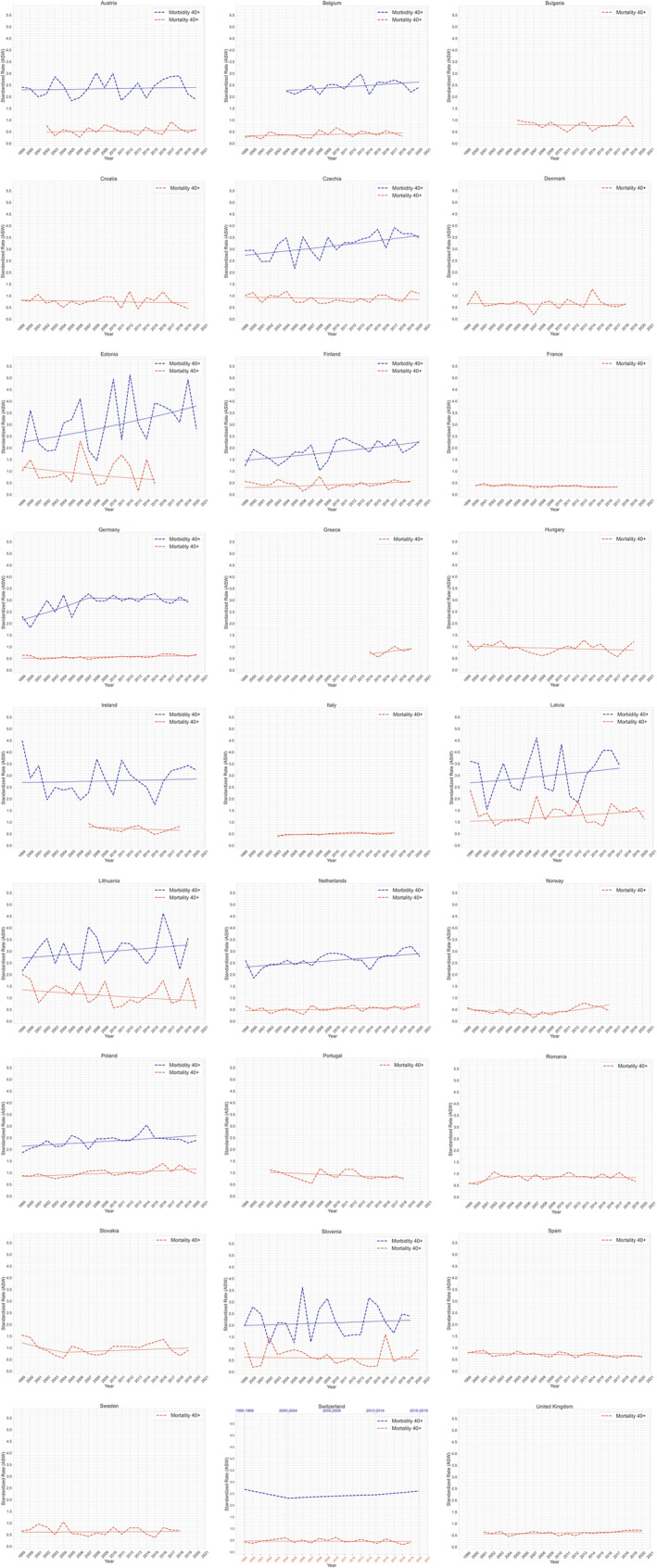
Trends in penile cancer incidence and mortality 40+.

**FIGURE 4:**
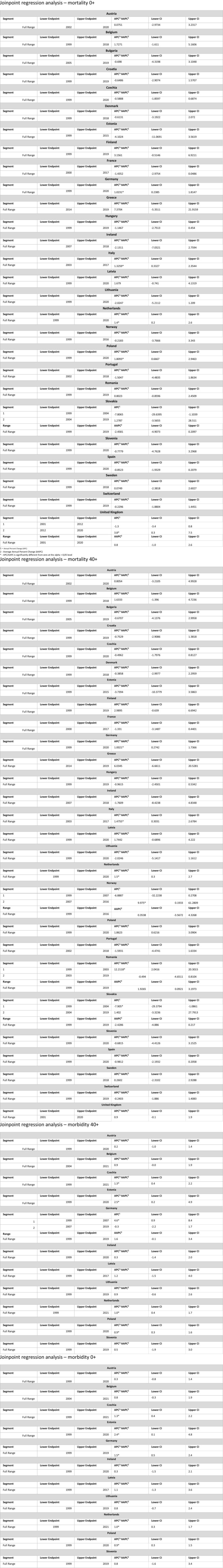
Joinpoint regression analysis—mortality and morbidity 0+ and 40+.

Time trends for individual countries were also observed in the 40+ age group (Figure 3). We evaluated trends in morbidity and mortality based on Jointpoint analysis, estimating the average rate of increase/decrease (Annual Percentage Change (APC), Average Annual Percentage Change (AAPC)). APC is a way of characterised trending cancer rates over time. This method assumes that cancer rates change consistently from the previous year. Annual changes in constant percentages change linearly on a log scale. AAPC is a summary of trends at pre‐defined fixed intervals. Using a single number, you can describe an average APC over several years. Even if the bindpoint model indicates trends that changed in those years, it is valid. It is calculated as an average weight of the APC of the corresponding point model, with a weight equal to the length of the APC interval.^4^, ^5^ Figure 4 presents the APC/AAPC tabular data for the 40+ and 0+ groups (as a reference group). Table 2 presents the proxy survival rates calculated for individual countries using the following formula (1−*M*/*I*) × 100, where *M* is the mortality (deaths) and *I* is the incidence (cases).^6^

**TABLE 2:** Proxy survival rates.

	Incidence	Incidence year	Deaths	Deaths year	Proxy survival
Austria	72	2020	20	2020	72%
Belgium	97	2020	15	2018	85%
Czechia	120	2020	40	2020	67%
Estonia	10	2020	4	2020	60%
Germany	954	2019	252	2020	74%
Ireland	43	2020	12	2018	72%
Latvia	17	2017	6	2020	65%
Lithuania	28	2019	4	2020	86%
Netherlands	182	2020	51	2020	72%
Poland	273	2020	110	2019	60%
Slovenia	19	2019	10	2020	47%

## 3 RESULTS

The relative distribution of penile cancer incidence and deaths in particular age groups is depicted in Figure 1. This information is presented collectively for all nations, based on the most recent year of analysis (Table 1). Penile cancer‐related morbidity and mortality rates have increased significantly after 40 years of age. It should be noted that the percentage of patients under the age of 40 is less than 2.5%. The majority of incidence cases and deaths (98.4% and 99.3%, respectively) occurred in people 40 years or older, with the highest incidence rates observed in those between 60 and 74 years. In the countries examined, there was an excess of deaths over incidence in the oldest age groups (75 years or older).

The spatial analysis revealed four classes of incidence. In the 40+ men's group, the highest incidence of penile cancer was recorded, with an ASIR value of 3.01‐3.56, in Lithuania, Latvia, and Ireland. Intermediate incidence values were found for Czechia, Belgium Germany, Estonia (ASIR 2.61‐3.01) and for Switzerland, Netherlands, Slovenia, Poland, and Finland (ASIR 1.88‐2.61). The lowest incidence was recorded for Austria (ASIR 1.88).

In the mortality analysis, we divided the countries into five classes (Figure 2B). In the 40+ age group, the highest mortality rate from penile cancer was observed in Czechia, Hungary and Latvia (ASMR 0.99‐1.22). Intermediate mortality values were recorded in Poland, Greece, Ireland, Slovakia and Slovenia (ASMR 0.74‐0.99), in Denmark, Germany, Sweden, Bulgaria, the Netherlands, Portugal, the United Kingdom, Romania (ASMR 0.6‐0.74), in Finland, Spain, Austria, Italy and Lithuania (ASMR 0.46‐0.60). The lowest mortality (ASMR 0.31‐0.46) was observed in Estonia, Norway, France, Belgium, Switzerland and Croatia.

We analyse trends in penile cancer incidence and mortality in age groups 0+ and 40+. Based on the results shown in Figure 1, we decided to present in Figure 3 trends for the age group 40+. The results of the time trends analysis for the 0+ group we have presented in Figure 4.

The incidence of penile cancer in both age groups (0+ and 40+ years) increased over the years analysed in Belgium, Czechia, Estonia, Germany, Latvia, Lithuania, the Netherlands, Poland and Slovenia. Morbidity rates for both age groups remained stable in Austria and Ireland. In Switzerland, we evaluated trends based on two 4‐year periods. The incidence in Switzerland decreased between the first two observation points, and then in subsequent periods showed an increasing trend until 2019, then returned (2015–2019) to the same ASW value as in 1995–1999.

Analysis of mortality time trends indicates a growing trend in the 0+ and 40+ groups in Germany, Italy, Belgium, Latvia, Poland, Finland, Greece (however, data for this country are available only from 2014 to 2019) and the Netherlands. In Romania, also in the first years of observation, there was an increase in mortality in both age groups; however, in 2003 this trend stopped in the 40+ group and the mortality rate showed a plateau. In the United Kingdom, there has been an increase in mortality, but this increase is statistically insignificant. In Austria, an increase in mortality was observed only in the 40+ year age group, while for the population covering all age groups, the trend remained stable.

In Sweden and Switzerland, the mortality trend remained stable in both the 0+ and 40+ age groups. In Norway, the curve for the 0+ group was stable, but there was a significant shift in the 40+ age group, from a decreasing trend to a rapid increase. In Denmark and in Czechia, a reduction in mortality was observed in the 0+ group and a stable trend was observed in the 40+ age group. In Estonia, Lithuania, Ireland, Portugal, France, Spain, Hungary, Croatia, Slovenia and Bulgaria, a decreasing trend in mortality was observed. In Slovakia, the downward trend continued until 2004, when a change occurred, and the mortality rate began to increase.

It was feasible to estimate a proxy for the survival rate for 11 of the 27 countries (owing to data accessibility). The highest survival rates were observed in Belgium and Lithuania, while the lowest survival rates were reported in Slovenia, as shown in Table 2.

## 4 DISCUSSION

Squamous cell carcinoma (SCC) is the predominant histological subtype of penile cancer, accounting for 95% of all cases, and the remaining 5% comprising sarcomas, melanomas or basaloid carcinomas. In some countries in Africa, Asia and South America, SCC is more common than in Western Europe and North America and accounts for up to 10% of all cancers. Factors such as socioeconomic conditions, sexual practices, religious practices (circumcision as a religious ritual) and the risk of exposure to human papillomavirus (HPV) infection may contribute to these differences. The penile glans, foreskin, the balanic groove and the penile shaft are the affected areas, with 48% of the cases occurring in the glans penis, 21% in the foreskin, 6% in the balanic groove and <2% in the penile shaft.^2^, ^7^, ^8^, ^9^

The main risk factors for penile cancer include phimosis, chronic inflammation of the glans penis and foreskin (e.g. associated with the presence of phimosis, lichen sclerosus), UVA phototherapy (the risk varies depending on the dose—at low doses it increases 16.3 times, and at high doses it increases by as much as 286 times), phototherapy with the use of psolarenes (e.g. due to psoriasis), smoking, HPV infection, low socioeconomic status, living in rural areas, early age of sexual initiation, high number of sexual partners and unmarried men (the incidence of penile cancer is lower among married men than among single men, as confirmed by studies conducted in Denmark as well as a study based on data from the United States, Canada and Europe).^2^, ^7^, ^8^, ^9^, ^10^, ^11^, ^12^, ^13^, ^14^

HPV infection occurs in approximately 30%–50%^2^ of patients with penile cancer and 70%–100% of patients with intraepithelial penile neoplasia.^2^ The most common types of HPV involved in penile carcinogenesis are 16 and 18.^2^ HPV infection has a variety of clinical manifestations, from asymptomatic to typical genital warts (HPV 6 and 11) and giant condyloma (Buschke‐Loewenstein tumour), which develop in non‐immunocompetent individuals.

The most important risk factor for the development of penile cancer, in addition to HPV infection, is the presence of phimosis. According to a study conducted in Brazil, phimosis was a risk factor in an average of 45% (25%–60%) of patients with penile cancer.^12^, ^13^ Furthermore, in both the United States of America and Brazil, it is believed that children who are circumcised at birth do not develop penile cancer. A 2011 meta‐analysis by Larke et al. showed that circumcision during childhood and adolescence protects against invasive penile cancer, with an overall odds ratio of 0.33 (95% CI 0.13–0.83).^12^, ^15^

Addiction to tobacco products is a widely recognised risk factor for the development of many cancers, including penile cancer. Smokers have a 2.4‐fold increased risk of developing penile cancer compared to non‐smokers.^8^, ^13^ The risk also increases with chewing tobacco.^10^ According to studies conducted in Sweden and India, the risk increases in smokers who smoke 10 cigarettes a day^9^, ^10^; however, American studies postulate a threshold of 20 cigarettes smoked per day.^10^ It is worth noting that the risk is also higher in ex‐smokers.^10^

People with a lower socioeconomic status are at increased risk of developing penile cancer. In the United States, the risk of penile cancer was 43% higher among men from regions where ≥20% of the population lived in poverty.^13^ The incidence also varies according to ethnicity; for example, the Hispanic‐American population living in the United States is 0.7/10^5^ people and in the Asian population it is 0.2/0^5^ people.^2^ The role of obesity as a risk factor for penile cancer is confirmed by a study that indicates an increase in the risk of the disease by 53% for every five units of increase in BMI.^13^ According to Zequi et al. penile cancer is more commonly found in patients with documented intercourse with animals.^16^

Prevention methods include HPV vaccination, the use of condoms to reduce HPV infections, smoking cessation, self‐examination as part of daily genital toileting, treatment of chronic penile inflammation, reduction of the use of photochemotherapy and personal hygiene.^2^, ^7^, ^10^, ^13^, ^15^ HPV vaccination is considered a method of prevention for penile cancer, but the percentage of men vaccinated against HPV worldwide is still low and research is needed on the effectiveness of including HPV vaccines in male vaccination programmes for the prevention of penile cancer and penile intraepithelial neoplasia.^17^ Circumcision is also a protective factor.

When evaluating the incidence of penile cancer in Poland, some under‐registration of cases can be expected. In the daily work of urologists, it is common practice not to perform a histopathological examination of resections obtained during circumcision. This problem does not only concern Poland. According to Pearce et al. histopathological examination of all foreskins obtained during circumcision is unprofitable. He estimates that of the 11,000 circumcisions performed annually in England, only 20% of resections are examined by a pathologist.^18^ Given the number of these procedures, it can be assumed that precancerous conditions and early forms of penile cancer remain undiagnosed.^19^ This is supported by another study conducted in England, where a retrospective study of 301 patients with lichen sclerosus revealed that 41 of them had the co‐occurrence of intraepithelial penile neoplasia.^16^, ^17^ In addition, intraepithelial penile neoplasia and penile carcinomas confined to the glans mucosa are often treated with topical methods, such as cryo‐ and electrodestruction, without taking representative specimens. Dispersion of the diagnosis and treatment of penile cancer among dermatologists and urologists negatively affects the completeness of the data. If a sample is taken from an area suspected of penile cancer, it is difficult to interpret because of the relatively rare occurrence of penile cancer. As Tang et al. rightly pointed out, reviewing 155 penile cancer biopsy samples that were referred from 15 regional centres to a reference centre, the definitive diagnosis was changed in up to 31% of cases, of which 60% of patients required a change in treatment.^19^

Only a few publications on this topic have evaluated the mortality of penile cancer in Europe. Moreover, the publications analysed the trends without explaining the reasons.^20^, ^21^, ^22^, ^23^, ^24^, ^25^

In England, researchers analysed trends in penile cancer mortality rates over a 31‐year period, from 1979 to 2009, using data from population‐based regional registries, where registration is voluntary and not mandatory. The study excluded carcinoma in situ from the analysis. Cohort mortality rate ratios were calculated for individuals born between 1890 and 1969 using indirect age standardisation. The findings revealed that mortality rates declined by 20% after 1994 (from 0.39 to 0.31 per 100,000) despite a rise in penile cancer incidence. The decrease in mortality was particularly evident for men born after 1915 and remained relatively stable for the more recent cohorts of men, indicating a genuine improvement in treatment efficacy in recent years in the England, which can be attributed to centralization.^20^

In a study conducted in Germany, researchers analysed mortality rates in Saxony from 1990 to 2012 using joinpoint regression. Data from the Federal German Statistical Office were used to investigate mortality trends. The results indicated that penile cancer mortality in Saxony exhibited a statistically significant negative trend during the period under investigation, with an annual percent change of −3.46 (95% CI −5.21 to −1.67).^21^

In Norway, researchers investigated the mortality trends of penile SCC over a 60‐year period spanning 1956–2015. Information was gathered from the Cancer Registry of Norway, and age‐standardised mortality rates were analysed using the annual percentage change statistic and joinpoint regression method. The results revealed an upward trend in the mortality rate during the study period.^22^

In Spain, a study was conducted to analyse mortality trends for HPV‐related cancers by gender from 1996 to 2010 and make predictions until the year 2025. The results of the study indicated stability in the penile cancer mortality rate, as determined by the joinpoint regression analysis. According to predictions made using the Nordpred program and the age–period–cohort model, it is expected that the penile mortality rate will increase until 2025.^23^

Over the last two decades (1997–2018), researchers in Denmark have analysed the mortality rates of penile SCC. This study utilised two high‐quality nationwide registries in Denmark, the Danish Cancer Registry and the Danish Pathology Registry, and found a decline in the penile cancer mortality rates.^24^

Similarly, a study conducted in the Netherlands between 1989 and 2006 examined the trends in penile cancer mortality. The data for this study were obtained from the Netherlands Cancer Registry, which has combined data from 8 Dutch regional cancer registries since 1989. Three‐year moving average age‐standardised mortality rates were calculated per 100,000 person‐years with standardisation performed according to the European standard population. Penile cancer mortality was evaluated by calculating the estimated annual percentage changes using the Joinpoint program, and the study showed a declining trend in mortality.^25^

Our research utilised data from the World Health Organisation Mortality Database to investigate trends in penile cancer mortality using the Jointpoint analysis. In the United Kingdom, we analysed data from 2001 to 2020 and observed a statistically insignificant increase in penile cancer mortality. In Germany, an increase in mortality rate was observed. In Norway, our findings are consistent with the conclusions of Hansen et al. as we observed a rapid increase in mortality among individuals aged 40 and over. In Spain, we observed a decreasing trend in mortality from 1999 to 2020, contradicting the predictions of de Souza et al. In Denmark, we observed a reduction in mortality among individuals aged 0–40 years in 2018, which is consistent with the findings of Olesen et al. and a stable trend among those aged ≥40 years. In the Netherlands, we observed a growing mortality trend among individuals aged 0–40 and 40 and over from 1999 to 2020, which contradicts the findings of Graafland et al. who analysed data from 1989 to 2006.^25^

In most of the European countries studied, there has been a declining trend in penile cancer mortality. In most of these countries, this trend remained consistent throughout the observation period. However, in some countries, such as Romania and Slovakia, this trend has changed over time. In 22 out of the 27 countries examined, the trend is consistent across both the age groups 40 and above and 0 and above. In contrast, in 5 countries, including Austria, Norway, Denmark, Czechia and Romania, the trend differs between these age groups. Notably, Poland stands out as an exception among European countries as it is experiencing an increasing trend in penile cancer mortality. This is attributed to a lack of recognition of cancer symptoms and inadequate attention to elderly patients by the healthcare system, as evidenced by the higher death toll among those aged 75 years or older.

Due to the rarity and complexity of the disease, the treatment of penile cancer is a diagnostic and therapeutic challenge for physicians, and the guidelines are based on a limited number of retrospective studies.^26^ Therefore, patients with penile cancer should be treated in specially selected centres, where urologists closely cooperate with radiologists, oncologists and pathologists dedicated to this disease.

According to Pecoraro et al. centralisation of penile cancer treatment results in an increase in penile‐sparing procedures (79.9% vs. 57.8%), invasive inguinal lymph node staging (90% vs. 41%), and more complete pathomorphological reports—especially with regard to the determination of p16, which is a marker of HPV infection (81.4% vs. 8.3%).^27^ Moreover, treatment of patients in highly specialised referral centres, where organ‐sparing surgery or invasive lymph node staging are used more frequently, increases survival (in the United Kingdom, in Norfolk County an increase in 5‐year survival of 7–12 percentage points).^26^

The centralization of health care for rare cancers is a positive development that should be promoted. In addition to the increase in survival, it also improves the quality of patient care through the exchange of knowledge and experience between centres and improves the accuracy of histopathology results. Centralised care was introduced in the United Kingdom (2002), the Netherlands and Scandinavia (Denmark, 2009). In the Netherlands, care for penile cancer patients is centralised in one national centre of expertise (Netherlands Cancer Institute, Amsterdam).^26^ In Denmark, care has been centralised to two university hospitals and two specialities, urology and oncology. In 2011, the Danish National Penile Cancer Quality Database (DaPeCa‐data) was established. This database records all newly diagnosed cases of penile cancer from June 2011 onwards and collects data prospectively.^26^

In Germany and Austria, the topic of centralising penile cancer care has been the subject of recent discussion. In January 2023, surveys were sent to 48 centres, and 75% of the respondents provided their input. In Germany, 94% of Urological University Department Chairs believed that centralisation was feasible in the medium term, while in Austria, 89% of Urological University Department Chairs held the same view. Among Urological University Department Chairs, 72% thought that centralization in university hospitals was appropriate, while 28% preferred a geographically orientated approach. Furthermore, 97% of the respondents suggested the establishment of penile cancer portals, which would provide a second opinion until centralisation was fully implemented.^28^

In Poland, efforts have been made to centralise the treatment of penile cancers. According to data from the PNCR, the Department of Urology at the Medical University of Gdańsk has seen a significant increase in the number of patients treated for penile cancer since 2019. When comparing data from 2011 to 2019 to the period of 2019–2023, there has been a significant increase in cases of penile cancer (from 45 out of 85 cases). During the period of 2019–2023, more than 90% of patients were treated with modern methods of penile sparing and lymph node staging. Unfortunately, 16 deaths from penile cancer occurred during the same period. This is a significant improvement compared to the period 2011–2019, when no organ‐sparing procedures were performed and 20 deaths occurred, resulting in a mortality rate of 44.4%.

In light of the issue of patients with penile cancer, the first Penile Disease Outpatient Clinic in Poland was opened in early 2020 at the Invasive Medicine Center of the University Clinical Center in Gdańsk. This clinic focusses on the diagnosis and treatment of penile diseases according to the guidelines of the urological and dermatological societies.^29^

Poland and most European countries follow the guidelines developed by the European Association of Urology (EAU) and the American Society of Clinical Oncology (ASCO) for the treatment of penile cancer. These guidelines were first published in 2000 and have been updated annually since 2010.

The choice of cancer treatment method depends largely on the stage of the disease. In the past, a 2 cm negative resection margin was required for penile tumours. However, the current guidelines recommend a safe negative margin of >1 mm.^30^ The approach to treatment for penile cancer has changed over time and organ‐sparing therapy is now considered the gold standard treatment.^31^ If penile‐sparing surgery significantly affects a patient's quality of life, then the involvement of regional lymph nodes, such as inguinal and pelvic lymph nodes, can determine survival. Therefore, patients with intermediate‐risk penile cancer (>T1aG2) and cN0 underwent invasive inguinal lymph node staging (dynamic sentinel or modified lymph node biopsy or inguinal lymph node dissection or radical inguinal lymph node dissection and pelvic lymphadenectomy), whereas cN+ patients underwent ilioinguinal lymph node dissection.^32^

In recent years, there has been a shift in the treatment of penile cancer. Research on immunotherapy^33^ and noninvasive node staging is ongoing.

Conventional systemic treatment for penile cancer involves the use of cisplatin and taxanes, with neoadjuvant treatment administered to patients who have large lymph nodes in the inguinal region or are undergoing pelvic lymphadenectomy. This approach aimed to increase the likelihood of successful surgical treatment. However, the efficacy of adjuvant chemotherapy remains debatable.

Although recent studies suggest that the use of cisplatin and taxanes in patients who have undergone inguinal or pelvic lymphadenectomy does not prolong overall survival,^34^ the European Association of Urology guidelines recommend that high‐risk penile cancer patients be discussed in a multidisciplinary group, which may include a urologist and an oncologist, as potential candidates for adjuvant chemotherapy.^35^

The findings generated by our research team are difficult to compare with previous results due to differences in the methodologies used. However, a comparison of the results of our analysis with those published in the past may provide insight into the trajectory of change.^36^ By categorising the countries into Western, Central and Eastern parts of Europe, the following analyses were conducted. For Western European countries such as Belgium (85%), the Netherlands and Ireland (72%), survival rates have improved when compared to the analysis conducted between 2002 and 2007 (66.3% for Western European countries). However, the survival rate for this type of cancer has decreased in Estonia (60%).

Among Central European countries, only Germany showed an improvement in survival rates for penile cancer (74%), while Austria remained the same (72%). Survival rates in the Czech Republic, Poland and Slovenia have deteriorated by 67%, 60% and 47%, respectively. In Eastern European countries, Lithuania and Latvia showed improved survival rates (86% and 65%, respectively), while the rate of this type of cancer decreased in Estonia (60%). In particular, the survival rate proxy is a reliable initial indicator for assessing the effectiveness of a country's healthcare system.^36^

The survival rate for men with organ‐limited disease is approximately 90% after 5 years. On the contrary, metastatic cancer has a marked decline in survival rates, dropping to approximately 80% for unilateral metastases in two or fewer lymph nodes, 10%–20% for bilateral lymph node involvement or pelvic lymph node metastases, and <10% for extranodal metastases. Patients who do not receive treatment typically die within 2 years of diagnosis.^2^, ^9^

Penile cancer is a highly aggressive disease with serious psychological and social consequences. Efforts are being made to increase public awareness of this disease and its impact on affected patients. The European Commission has established 24 European Reference Networks for rare diseases, one of which is eUROGEN, led by the EAU, in the field of urology.

Due to the limited availability of data on penile cancer from national registries, there are few studies on the incidence of this cancer. Our study is the first to cover the incidence of penile cancer in many European countries and estimates an approximate survival rate for large populations, which is rarely cited in the literature. However, our study has some limitations, such as the short time period analysed (1999–2020), and the limited data availability of the national cancer registries. Moreover, the survival rate proxy used in our research is only an approximation of real values, depending on the availability and quality of data; however, it seems adequate in this study given the amount and quality of the available data. Creating a penile cancer registry in Poland, similar to Denmark, could lead to innovative evidence‐based solutions also for Polish patients. Centralising the healthcare system for rare cancers, as seen in countries such as the Netherlands, Germany, Denmark and the United Kingdom, is a positive development that should also be promoted in other European countries, including Poland.

## 5 CONCLUSIONS

Despite its rarity, penile cancer has piqued the interest of epidemiologists. Throughout the examined period, the incidence of penile cancer increased in the countries considered, and the trend in Poland was consistent with the European trend. However, underreporting of penile cancer was detected, and the higher mortality rate among individuals aged 75 years or older might indicate a failure to recognise cancer symptoms and a disregard for older individuals by the healthcare system. Unlike most European countries, Poland is experiencing an increasing trend of penile cancer mortality.

## AUTHOR CONTRIBUTIONS

**Iwona Wnętrzak:** Main conceptual ideas, data collection, review of the literature, project administration, wrote and edited the manuscript with input from all authors. **Mateusz Czajkowski:** Conceptualization, developed the outline and project plan, critical revision of the manuscript for important intellectual content. **Klaudia Barańska:** Acquisition, analysis, and interpretation of data, statistical analysis, visualization of results, critical revision of the manuscript for important intellectual content. **Marta Miklewska:** Acquisition, analysis, and interpretation of data, statistical analysis, critical revision of the manuscript for important intellectual content. **Urszula Wojciechowska:** Acquisition, analysis, and interpretation of data, critical revision of the manuscript for important intellectual content. **Roman Sosnowski:** Review of the literature. **Joanna A. Didkowska:** Devised the project, the main conceptual ideas and proof outline, critical revision of the manuscript for important intellectual content. Senior author.

## CONFLICT OF INTEREST STATEMENT

The authors declare no conflicts of interest.

## Data Availability

Data available on request from the authors. The data that support the findings of this study are available from the corresponding author upon reasonable request.
